# The pattern of c-Fos expression and its refractory period in the brain of rats and monkeys

**DOI:** 10.3389/fncel.2015.00072

**Published:** 2015-03-12

**Authors:** Vanessa N. Barros, Mayara Mundim, Layla Testa Galindo, Simone Bittencourt, Marimelia Porcionatto, Luiz E. Mello

**Affiliations:** ^1^Department of Physiology, Universidade Federal de São PauloSão Paulo, SP, Brazil; ^2^Department of Biochemistry, Universidade Federal de São PauloSão Paulo, SP, Brazil

**Keywords:** c-Fos expression, brain plasticity, marmosets, phylogeny, neuronal activation

## Abstract

Intense activation of neurons triggers the appearance of immediate expression genes, including c-Fos. This gene is related to various signal cascades involved in biochemical processes such as neuronal plasticity, cell growth and mitosis. Here we investigate the expression pattern and the refractory period of c-Fos in rats and monkey’s brains after stimulation with pentylenetetrazol. Rats and monkeys were sacrificed at various times after PTZ-induced seizure. Here we show that rats and monkeys already showed c-Fos expression at 0.5 h after seizure. Yet, the pattern of protein expression was longer in monkeys than rats, and also was not uniform (relative intensity) across different brain regions in monkeys as opposed to rats. In addition monkeys had a regional brain variation with regard to the temporal profile of c-Fos expression, which was not seen in rats. The refractory period after a second PTZ stimulation was also markedly different between rats and monkeys with the latter even showing a summatory effect on c-Fos expression after a second stimulation. However, assessment of c-Fos mRNA in rats indicated a post-transcriptional control mechanism underlying the duration of the refractory period. The difference in the protein expression pattern in rodents and primates characterizes a functional aspect of brain biochemistry that differs between these mammalian orders and may contribute for the more developed primate cognitive complexity as compared to rodents given c-Fos involvement in cognitive and learning tasks.

## Introduction

At the basic functional level, neurons, regardless of whether in rodents or primates show a remarkable similarity in their biochemical and biophysical characteristics. Indeed at the nervous system level, the few main differences described between rodents and primates concern most notably brain and neuronal size in addition to the distribution of specific neuropeptides (Finlay and Darlington, 1995; Clark et al., 2001; Finlay et al., 2001; Pearce et al., 2013). Therefore, broad differences at the biochemical level that could have wide implications for the understanding of different brain functioning between rodents and primates have not been reported, with the exception of those related to specific neuronal systems (e.g., visual and olfactory).

One of such fundamental biochemical elements is the proto-oncogene c-Fos which encodes a nuclear phosphoprotein Fos, that forms a dimeric complex with another protein named Jun, that exhibits a DNA sequence binding sites for the transcription factor activator protein-1 (AP-1). JUN family and Fos-related proteins forms countless possible combinations among its members, controlling other late genes and proteins related to cognition, motor and learning processes (Morgan et al., 1987; da Silveira et al., 2007). Several studies with mice and rats showed a seemingly similar expression of c-Fos in several regions of the central nervous system when the animal is exposed to a wide range of stimuli (water stress, fear, odors, intraparenchymal injection of various substances, including convulsing agents Dragunow and Robertson, 1987; Le Gal La Salle, 1988; Simler et al., 1994; Szyndler et al., 2009; Kawashima et al., 2014).

It is well known that rodents express c-Fos after seizure (Dragunow and Robertson, 1987; Le Gal La Salle, 1988; Simler et al., 1994). Preliminary evidence from our laboratory indicated that the temporal expression patterns of c-Fos after similar seizure events differed between rats and marmosets. One of the potential reasons would be a different seizure profile in terms of its temporal progression or intensity between these 2 different species. Yet our data concerning all of the observable behavioral features of seizure expression after PTZ (pentylenetetrazol) in rats and marmosets indicate no significant differences on that regard (Bachiega et al., 2008; Blanco et al., 2009). Therefore, this paper aims to draw a profile of expression of c-Fos by stimulation with PTZ in rats and monkeys in various regions of the central nervous system in order to test the possible differences that may exist between these two species.

## Material and Methods

### Subject

To determine whether the expression of immediate early genes would constitute one of such basic functional differences between rodents and primates we assessed its expression after seizures in rats and marmosets.

We used 50 marmosets (*Callithrix jacchus*) and 50 rats (*Wistar)* males and females weighing 250–400 g aged between 8 to 9 weeks in rats and 1–2 years in marmoset. The protocols used were approved by the Ethical Committee of Use and Care of Animals of UNIFESP (CEP No. 0175/12) and by the Ministry of Environment of Brazil. In experiment 1, which aimed to define the expression pattern of c-Fos in both species, we obtained the following groups (*n* = 5/group): Control group–corresponding to animals anesthetized and sacrificed without administration of any vehicle/drug except anesthetics; sal 1 h: corresponding to those animals that were anesthetized and sacrificed with administration of saline (only rats); PTZ 0.5 h: animals that received PTZ and 30 min after the seizure were sacrificed; PTZ 1 h: animals that received PTZ and 1 h after seizure were sacrificed and so on in PTZ 2 h (only in rats), PTZ 3 h (rats and marmoset), PTZ 6 h (rats and marmosets), PTZ 9 h (marmosets) and PTZ 12 h (marmosets only). In experiment 2 aimed at investigating the refractory period the groups were (*n* = 5/group): in rats (groups PTZ 1 h/1 h; PTZ 3 h/1 h and PTZ 6 h/1 h) and in marmosets (PTZ 3 h/3 h; PTZ 6 h/3 h and PTZ 12 h/3 h). The first hour refers to the application time of the second dose of PTZ after the first seizure, and the second hour refers to the time that they were sacrificed after the second seizure.

In this work (both for Experiment 1 and Experiment 2), we considered an animal to have had seizures only if the animal reached stage V of the Racine scale (1972). As for the time used for composing the different experimental groups, we started counting the time just after the animal reached stage V or after administration of saline (in case of sal 1 h group). The latency for reaching stage V seizures, was in the range of 1–2 min in rats and 1–5 min in monkeys. A detailed report of PTZ-induced seizures in marmosets can be found elsewhere (Bachiega et al., 2008). For all means the seizures induced in rats and marmosets were behaviorally similar in latency, intensity and duration.

### Immunohistochemistry Analysis

The animals were subjected to intraperitoneal injections of PTZ (50 mg/kg) or saline and sacrificed at specific time points. All animals were anesthetized with ketamine (60 mg/kg) and xylazine (15 mg/kg) intraperitoneally and subsequently decapitated. The right hemisphere was stored −80°C and later used for molecular biology procedures. The left hemisphere was placed in Eppendorf tubes (30 mL) containing 4% paraformaldehyde in PBS 0.01 M, pH 7.2, at 4°C for 5 days and then dried for 2 or more days in 30% sucrose in PBS 0.01 M pH 7.2 at 4°C for subsequent immunohistochemical analysis for c-Fos.

After fixation and dehydration the left hemispheres were sectioned in a cryostat in coronal sections of 30 µm thickness. The sections were processed for the immunohistochemical detection of c-Fos protein using a conventional avidin–biotin–immunoperoxidase technique to localize an antiserum raised against a synthetic N-terminal fragment of human Fos protein (rabbit polyclonal ab-5, Calbiochem). Briefly, free-floating sections were pretreated with hydrogen peroxidase for 10 min. Sections were treated with normal goat serum (1:100) and 0.3% Triton X-100 for 2 h and incubated with the primary antiserum at a dilution 1:5000 in KPBS at room temperature for 24 h. Subsequently, the sections were incubated with a secondary antibody (goat anti-rabbit IgG 1:200—Vector) for 2 h at room temperature and treated with avidin–biotin complex (Vector 1:100) for 90 min. Sections were submitted to nickel-intensified diaminobenzidine reaction. Between steps, the sections were rinsed in KPBS (pH 6.8) 0.05 M. The tissue was agitated on a rotator between each incubation and rinse step. Sections were mounted on gelatin-coated slides, dried, dehydrated, and coverslipped. To avoid eventual bias, at least one animal from each group was included in every staining batch. Four sections for each region (cingulate gyrus, piriform and primary motor cortex) were mounted on slides for histological evaluation. Histological counting of cells expressing c-Fos protein in the brain regions of interest was performed using the ImageJ® 1.45 s (National Institutes of Health USA) (Carnevali et al., 2004).

The number of c-Fos-positive cell nuclei within each area was counted in four consecutive sections per animal and the average of them was expressed as number of c-Fos-positive cells × 10^−5^/µm^3^. Stereological methods were not employed in this study due to potential bias associated with counts generated in this manner, such as uncertainties as to the extent to which antiserum penetrates through the thickness of the tissue sections and difficulties in defining the boundaries of the several cell groups of interest. Moreover, our interest was to make only relative comparisons of the strength of Fos induction as a function of the treatment status (Li and Sawchenko, 1998; Medeiros et al., 2003).

### qPCR Analysis

The right hemispheres were removed from the −80°C and homogenized in TRIzol® Reagent (Invitrogen) (2 mL for rats, 7 mL for marmosets) with the aid of ULTRA80 hand homogenizer (Ultra Stirrer) with stem 10 mm, in solution. The purified RNA (as TRizol protocol) was ressuspended at 202 µm of sterile water at 60°C, and 2 µL of this solution was used for quantification in a spectrophotometer (ND-1000 NanoDrop Technologies, Wilmington, DE, USA) for subsequent cDNA synthesis. After quantification, 2 µm of total RNA were used for synthesis of the complementary DNA strand. To this was added 1 µL RNA Oligo (dT) 15 primer (Promega, Madison, WI, USA) and RNase free H_2_O to complete 5 uL volume. This mixture was incubated for 5 min at 70°C followed by 5 min at 4°C. After this incubation period, we added 0.5 µL RNasin® Ribonuclease Inhibitor (Promega), 1.0 uL of 10 mM deoxynucleotide triphosphate (dNTP Promega), 1.0 uL IMPROM–II Reverse Transcription System (Promega samples to ), 4.0 µL of enzyme buffer, 2.4 µL of 25 mM MgCl_2_ and 6.1 uL H_2_O RNase-free. The conditions used for amplification in a thermocycler (Eppendorf) were: 25°C for 5 min, 42°C for 60 min, 70°C for 15 min. After synthesis, the c-DNA samples were stored at −20°C. To verify the efficiency of RT-PCR, a reaction of conventional PCR was performed for amplification of the c-Fos.

The qPCR reaction was performed using Brilliant® II SYBR® Green QPCR Master Mix (Stratagene, La Jolla, CA, USA) in a thermocycler Stratagene Mx3000P QPCR System (Stratagene) (Livak and Schmittgen, 2001). The c-fos primer for rats is forward 5’ ACGGAGAATCCGAAGGGAAAGGAA 3’ and reverse 5’ TCTGCAACGCAGAC TTCTCGTCTT 3’. The hprt gene was used as endogen primer (rats: forward 5’ CTCATGGACTGATTATGGACAGGA C 3’ reverse 5’ GCAGGTCAGCAAAGAACTTATAGCC 3’). The expression of c-Fos was normalized to hprt expression and calculated using the 2-ΔΔCt method [13]. Data analysis was performed with the StatView® for Windows software version 5.0.1. Comparisons between data were made by ANOVA followed by Fisher test. The level of significance was set at *P* < 0.05 and *P* < 0.001.

## Results

### The Expression Pattern

**Figure 1:**
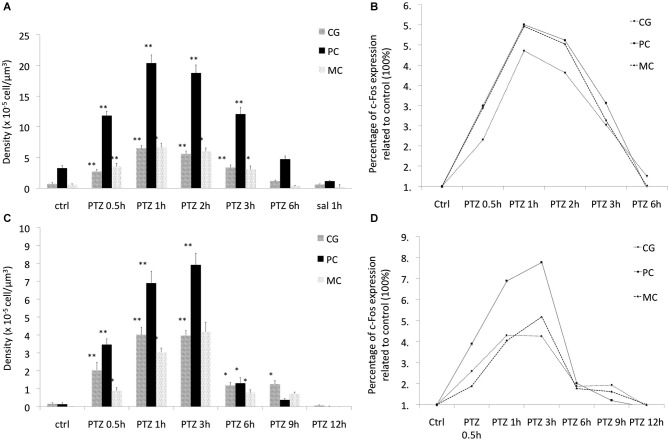
**The expression pattern of c-Fos in rats and marmosets. (A)** The expression pattern of c-Fos in cingulate gyrus (CG), piriform cortex (CP) and motor cortex (MC) in rats. **(B)** The relative expression of c-Fos in each of the assessed regions in rats, **(C)** the expression pattern of c-Fos in CG, piriform cortex (PC) and MC in marmosets. **(D)** The relative expression of c-Fos in each of the assessed regions in marmoset. *p < 0.05 **p < 0.001.

**Figure 2:**
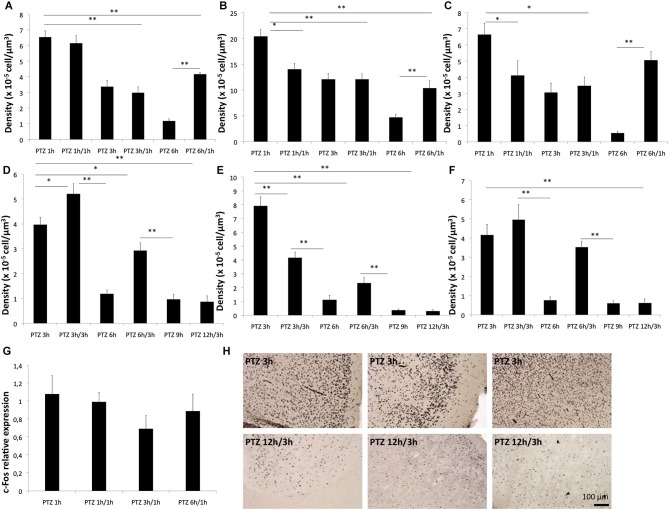
**The refractory period of c-Fos**. The refractory period of c-Fos protein in CG **(A,D)**, piriform cortex **(B,E)** and MC **(C,F)** respectively in rats **(A–C)** and marmosets **(D–F)**. Relative expression of c-Fos mRNA in fold change is shown in **(G)**. Representative photomicrographs of the c-Fos immunoreactivity for the refractory period assessments for a in marmosets are shown in H, compare the expression pattern at 3 h (after a single seizure) with that 3 h after a second PTZ seizure (12 h after the initial seizure) respectively for cingulate, piriform and MC. *p < 0.05 **p < 0.001.

**Figure 3:**
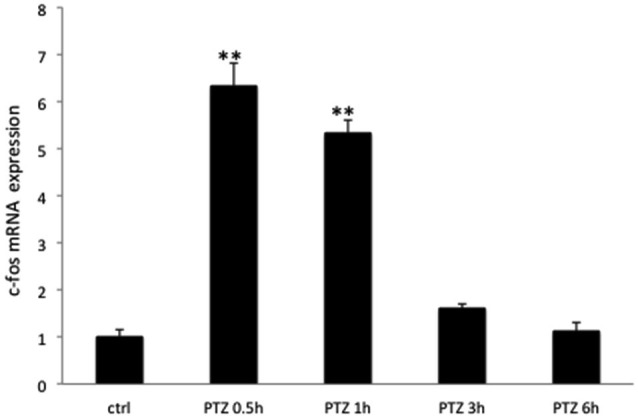
**The relative expression pattern of c-Fos mRNA after PTZ seizures in rats**. The expression of c-Fos mRNA in groups from Experiment 1 (Control, PTZ 0.5 h; PTZ 1 h, PTZ 3 h, PTZ 6 h) of rats. *p < 0.001.

As expected seizure induction by means of PTZ lead to increased expression of c-Fos at the immunocytochemical level in rodents already at 30 min (Szyndler et al., 2009). Maximum expression levels were observed at 1 and 2 h after seizure induction, returning to baseline values at 6 h. This same expression pattern was observed in all of the 3 brain regions assessed (Figures 1A, 2). c-Fos mRNA levels were at the maximum expression level 30 min after seizures, and had returned to baseline values after 3 h (see Figure 3). To this end our results match those previously reported after stimulation with metrazol, a similar convulsing agent, and also with PTZ (Sonnenberg et al., 1989; Clark et al., 2001). Our assessment of mRNA for c-Fos in marmosets did not yield results. It is likely that degradation of the samples influenced our ability to evaluate the marmosets c-Fos mRNA.

To determine whether this pattern would be conserved in primates we then investigated c-Fos expression in marmosets under the same stimulus parameters. As compared to rats, marmosets had expression of c-Fos notably longer than rats in all assessed brain areas. Even though with a similar initial surge at 30 min after PTZ seizure induction, c-Fos expression was markedly above control levels still at 6 h (Figure 2). Indeed, at 6 h after seizures, when c-Fos had returned to basal levels in rats, marmosets still exhibited 200% more than basal levels (Figures 1B,D). Return to baseline levels was only seen after 9 h in piriform and motor cortex (MC) and after 12 h in the cingulate gyrus (CG; Figures 1C,D, 2).

Unlike rodents, there was a greater topographic specificity of the expression pattern of c-Fos in the evaluated brain areas (with each brain area exhibiting different temporal profile of c-Fos expression), which allows speculation about a greater complexity (less stereotypy) of the biochemical processes mediated by c-Fos in marmosets as compared to rats. In addition, peak c-Fos expression in marmosets ranged from 400% to 800% as compared to basal levels while in rats c-Fos peak was rather homogeneous for the evaluated brains regions and did not exceed 500% (see Figures 1B,D).

### The Refractory Period

After an initial stimulus c-Fos expression has been reported to show refractory period to subsequent stimulation of similar nature (Morgan et al., 1987). For many, this has also been associated with c-Fos expression having a link to novelty, i.e., c-Fos would be also a marker of functional change and adaptation (Hoffman and Lyo, 2002).

The second seizure induced by the second dose of PTZ was generally similar to seizure generated by the first dose of PTZ. However, the time between application of PTZ and the seizure (seizure latency) did show some variation from animal to animal and was shorter in experiment 2 (about 30 s [range 15–120 s] in rats and 1 min [range 45–420 s] in monkeys) in relation to the experiment 1 (1–2 min [range 50–800 s] in rats and 5 min [range 140–720 s] in monkeys). A few marmosets exhibited prolonged seizure in experiment 2 (lasting 1 h or more), these animals were excluded from the analysis. The average duration of a stage V seizure was 46 ± 26 s for rats and 68 ± 23 s for marmosets, after a single seizure induction. The average duration of the stage V seizure for a second seizure induction 1, 3 and 6 h after the initial seizure induction ranged from 44 to 62 s for rats. The average duration of the stage V seizure for a second seizure induction 3, 6 and 12 h after the initial seizure induction ranged from 58 to 74 s for marmosets. The duration of the second seizure induction for rats or marmosets did not differ from that of the first seizure induction.

**Figure 4:**
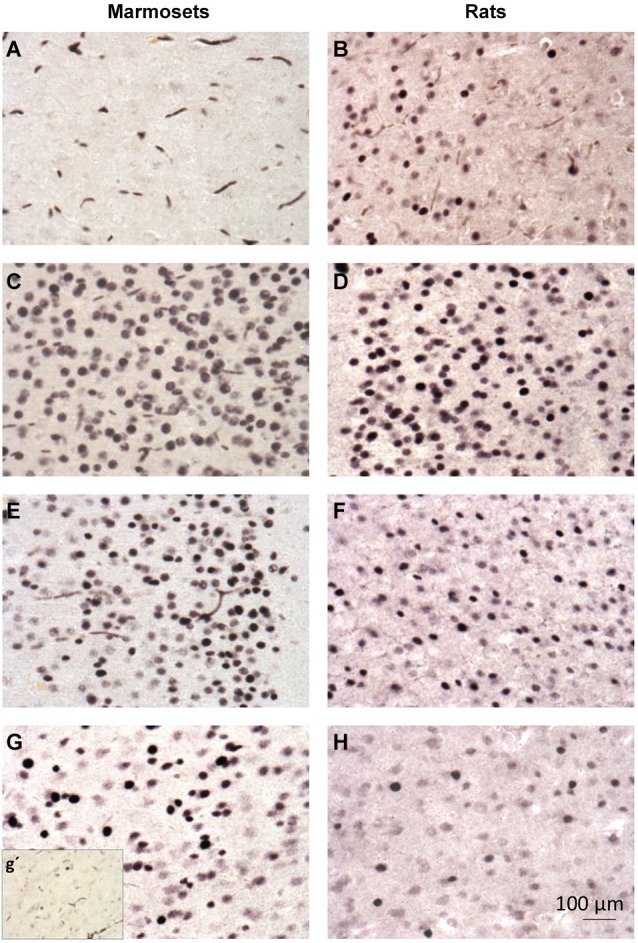
**c-Fos immunohistochemistry in the cingulate cortex of marmosets (first column; A,C,E,G) and rats (second column; B,D,F,H). (A,B)**—control group; **(C,D)**- PTZ 1 h; **(E,F)**- PTZ 3 h; G-PTZ 9 h; g’- PTZ 12 h; H- PTZ 6 h.

We next sought to determine whether the refractory period of c-Fos (for a second stimulus of the same nature of the initial one) was also different between rats and marmosets as a function the different temporal profile of expression seen after a single stimulus. Here, we found that for the cingulate cortex of rats, there was no refractory period at 1 h and there was an absolute refractory period at 3 h. In other words, a second seizure induced in rats, 3 h after the first one, did not trigger any change in protein levels of c-Fos in the cingulate cortex. At 6 h after the first seizure rats showed a relative refractory period, meaning that a second seizure induction 6 h after the first one had a partial effect in the CG c-Fos expression in rats (Figure 4A). In the piriform cortex of rats, there was an absolute refractory period at 3 h and relative refractory period at 6 h while in the MC we observed an absolute refractory period at 1 h and 3 h, and no refractory period at 6 h (Figures 4B,C).The mRNA levels showed no significant differences between the groups that had a refractory period and PTZ 1 h (single induction), suggesting a post-transcriptional mechanism of induction of the refractory period (Figure 4G).

For the marmosets in contrast the CG of rats we observed a refractory period at 6 and 12 h, but no refractory period at 3 h (Figures 4D–F). Strikingly for marmosets a second seizure induced at 3 h after the first seizure lead to an even significantly greater c-Fos level than those of the PTZ 3 h group (single induction). Therefore, for marmosets even at the peak expression of c-Fos, a second stimulus was able to sum over the first. This feature of stimulus adding was not found in any of the three brain areas evaluated in rodents. Unlike the CG, the piriform cortex of monkeys exhibited refractory periods at 3, 6 and 12 h, in MC of monkeys there were no refractory period except at 12 h (Figure 4H).

## Discussion

### The c-Fos Expression Pattern

Our study shows an increase in the mRNA expression after 0.5 h of PTZ-induced seizure in rats and declining expression levels after 1 h, returning to basal levels 6 h after seizure. Other studies (Ikeda et al., 1990; Chaudhuri, 1997; Chaudhuri et al., 2000) indicate that c-Fos mRNA expression in fact may be above baseline levels already after 15 min of stimulation. Stimuli such as dehydration (da Silveira et al., 2007) tactile exploration (Melzer and Steiner, 1997; Bisler et al., 2002) visual stimulation (Baille-Le Crom et al., 1996), mechanical trauma (Buytaert et al., 2001), encephalic lesion (Schreiber et al., 1993), stress (Cullinan et al., 1995) showed different c-Fos expression patterns than those with PTZ stimulation.

Sonnenberg et al. (1989) showed elevated mRNA levels of c-Fos at 30 min and 60 min after stimulation with PTZ in rats. This returned to baseline levels 4 h after the seizure, whereas in our studies, mRNA levels had significantly diminished by 3 h (we did not evaluate at 4 h) and returned to baseline after 6 h. One of the original reports of c-Fos expression after seizures showed similar expression pattern in rats as that reported above and in addition indicated that after an initial return to baseline levels 3 h after seizure, c-Fos levels showed further decrease and returned to baseline levels only 16 h after seizures (Morgan et al., 1987).

In our study, we observed a longer refractory period in primates as compared to rodents in all studied areas. The cingulate cortex was notably the one area standing out in this respect. Surprisingly, with the second stimulus marmosets, but not rats, showed an ability to increase c-Fos expression above the initial peak (due the initial first seizure). There are reports indicating that not all stimuli lead to a refractory period for c-Fos expression. Indeed lithium administration 20 min or 1 after an initial administration result in c-Fos expression (Spencer and Houpt, 2001).

One of the few studies that attempted to map the temporal pattern of expression of c-Fos (Kazi et al., 2004) assessed its levels in the nuclei habenulares in which primates were enucleated, showing an increased expression bilaterally within 1 h increasing and decreasing until 6 h after reaching baseline c-Fos after 27 h. Consistently, studies with enucleation in rats (Gonzalez et al., 2005) show the same temporal pattern of longer lasting expression of c-Fos. One may speculate that as enucleation is an enduring (and not a temporally discrete) stimulus, likely associated with progressive loss of neuronal connections in several brain areas, it may lead to a more prolonged pattern of c-Fos expression.

### Possible Function Significance

It is known that the better the integration between different brain areas the more complex can be the associated behavioral responses. This can be seen for example, in a cognitive task as discrimination of objects, when the association between higher processing areas of visual, tactile and mnemonic information provoke better animal performance during task performance (Urcelay and Miller, 2014). Our hypothesis is that the expression of c-Fos in these brain areas during learning might be one of the mechanisms of integration of information. As a longer time interval is available, the greater the chance for subsequent association between different stimuli activating different regions of the central nervous system.

Understanding the refractory period has tremendous implications for our understanding of consolidation. This has been address, for example, by Guzowski et al. (2006) in rodents using the immediate early gene Arc. Extrapolating from this work and our own data, it may be that the spacing of trials to achieve the most efficient learning is much longer in the monkey than rodent.

There have been reports of specific biochemical differences in discrete neuronal systems between primates and rodents or humans and non-humans primates. These include the extent of cortical synapses (Liu et al., 2012) connections of the striatal dopaminergic system (Berger et al., 1991) form and function of the hippocampus (Clark and Squire, 2013), the organization and size of pyramidal cells (Elston and Manger, 2014), gene expression and rearrangement of genes in chromosomes (King and Wilson, 1975).

We provide direct evidence that marmosets (and likely most primates) have a greater complexity in the neuronal activation of different brain areas as a function of stimulation as compared to rats (and presumably most rodents). The increased temporal window of c-Fos expression, in marmosets as compared to rats, discovered in this study may serve important functions in ensuring distinctive characteristics of learning and memory processes in primates.

Less stereotyped biochemical processes (as shown here) as well as greater windows for temporal integration of events and a more complex pattern with regard to summation or refractoriness, all allow for greater functional capacity of the nervous system of marmosets as compared to rats. For example, in a cognitive task such as the discrimination of objects, when there is association between higher processing areas of visual, tactile and mnemonic information there is better animal performance during task performance (Spencer and Houpt, 2001). Our data allow us to speculate that major functional differences between the cognitive abilities of rodents and primates might rely on similar differences in the temporal profile of a various biochemical steps.

## Author Contributions

VNB., as the principal author, did all experimental procedures (was involved with project design, application of PTZ or saline, histochemistry, cell counting, PCR real time, analyzed data, statistical analysis and wrote the paper). MM, LTG and MM helped with PCR real time procedures, primers design and approval the version to be published. SB was involved with designed project and helped with immunohistochemistry. LEM. designed the project, coordinated the group, and wrote the paper with VNB and final approval the version to be published.

## Conflict of interest statement

The authors declare that the research was conducted in the absence of any commercial or financial relationships that could be construed as a potential conflict of interest.
